# Does the high dietary diversity score predict dietary micronutrients adequacy in children under 5 years old? A systematic review

**DOI:** 10.1186/s41043-022-00337-3

**Published:** 2023-01-06

**Authors:** Roghayeh Molani-Gol, Sorayya Kheirouri, Mohammad Alizadeh

**Affiliations:** 1 Student Research Committee, Tabriz University of Medical Sciences, Tabriz, Iran; 2 Department of Nutrition, Faculty of Nutrition and Food Sciences, Tabriz University of Medical Sciences, Attar Nishabouri St., POBOX: 14711, Tabriz, 5166614711 Iran

**Keywords:** Dietary diversity, Micronutrient adequacy, Micronutrient deficiency, Infants, Children

## Abstract

**Backgrounds:**

Undiversified and monotonous diets can lead to deficiency disease, named micronutrient deficiency, more specifically among young children. Dietary diversity (DD) has been known as a valid indicator to assess micronutrient inadequacy of the diet. The aim of this study was to determine “is there an association between high dietary diversity and the micronutrient adequacy, in children under 5 years old?”.

**Methods:**

PubMed, Scopus, ScienceDirect, Web of Sciences, and Google Scholar databases were searched until February 2022, without date restrictions, using relevant keywords. All original articles, written in English, evaluating the relationship between DD and micronutrient adequacy in children under 5 years were eligible for this review.

**Results:**

Totally, 1814 records were found in electronic search databases; after removing duplicated and irrelevant studies according to the title and abstract, the full text of the 35 articles was critically screened, in which 15 cross-sectional studies were included in this review. All of these studies reported that DD of infants and children under 5 years was positively associated with their micronutrient adequacy.

**Conclusion:**

The findings indicate that in infants and children under 5 years, intake of various food groups reveals the adequate intake of micronutrients.

**Supplementary Information:**

The online version contains supplementary material available at 10.1186/s41043-022-00337-3.

## Background

Micronutrients are the essential vitamins and minerals required to sustain almost all normal cellular and molecular functions [1]. Micronutrient deficiency (MND) is defined as the percentage of individuals whose micronutrient intake is less than the estimated average requirement [2]. While the micronutrient required amounts are minimal, MND can have several negative impacts on health such as developmental problems that if left untreated will ultimately lead to death [3]. MND is coming to be the most prevalent nutritional deficiency [4] that based on World Health Organization (WHO) reports affects an estimated 2 billion people in the world [5]. The prevalence of MND has increased in the early stages of life due to poor feeding during this period and children under 5 years of age and younger are at the highest risk and one of the most vulnerable population subgroups [6, 7].

The nutritional quality of infants and young children is a public health concern in the world [8]. These age-groups require diets with high nutrient density and variety because of their rapid growth and development [9]. Inadequate intake of micronutrients is primarily responsible for the deficiencies that are due to poor quality of diet and monotonous diets [10, 11]. Dietary diversity (DD) is a good predictor of dietary quality and has been known as a valid indicator to assess micronutrient inadequacy of the diet, more specifically among infants and young children, by FAO [12–14]. Assessment of DD helps to determine whether the child’s diet has the important elements needed for growth. Intake of a wide variety of foods increases micronutrient adequacy that is necessary for better growth and proper nutrition of children [15].

Many studies have reported that children's diets are rich in energy-dense micronutrient-poor foods and they are not eating enough fruit and vegetables [8, 16, 17]. Children under 5 years of age remain the most vulnerable group to malnutrition and micronutrient deficiency as chronic malnutrition. To our best knowledge, there is no systematic review study evaluating the relationship between DD and micronutrient adequacy in infants and young children. Hence, the research question is that “Does high dietary diversity scores predict dietary micronutrient adequacy in children under 5 years old? Therefore, this systematic review was carried out to summarize the present evidence of the relationship between DD and micronutrient adequacy in children under 5 years.

## Method

The protocol of the study was registered and approved by the Research Vice-Chancellor of Tabriz University of Medical Sciences (Research ID: 66585).

### Data sources and search strategy

The present systematic review was conducted based on Preferred Reporting Items for Systematic Reviews and Meta-Analyses (PRISMA) guidelines [18] by focusing on the relationship between DD and micronutrient adequacy. This study was performed using the related keywords (“Dietary diversity or diet diversity or food diversity or diet variety or dietary variety or food variety” and “micronutrient*” and “children or infant”) on the electronic databases including PubMed, ScienceDirect, Scopus, and Google Scholar. Moreover, to ensure the inclusion of all eligible studies, forward and backward citation was tracked for all of the included studies. The search was limited until February 2022. The method of the database search strategy is shown in Additional file 1: Table S1.

### Eligibility criteria and screening method

Articles published in the English language that examined the relationship between DD and micronutrient adequacy in infants or children under 5 years were included in the present study. The exclusion criteria were book chapters, conference abstracts, letters, posters, editorial, commentary, thesis, and review articles. Moreover, articles that studied the relationship between household DD and micronutrient adequacy or performed on other age-groups, and studies that their full text was not available were excluded from this review.

After removing the duplicate studies using the EndNote software (Version X9; Thomson Reuters, Philadelphia, PA, USA), two independent investigators initially screened titles and abstracts of the searched studies based on the inclusion and exclusion criteria. In the second step, the full text of papers that were eligible was assessed and studies that had sufficient information or could meet the predefined criteria were included in this review.

### Data extraction

The following data were extracted from selected studies: the first authors' name, year of publication, study location and design, age of children, sample size, method and duration of food intake assessment, number of considered food groups, classification of DD, DD and micronutrient density adequacy (MDA) criteria, adjusted covariates, findings with respect to the relationship between DD and micronutrient adequacy, and *p* value.

### Assessment of articles’ quality and risk of bias

The quality of the selected articles was assessed by the two independent authors using the d for cross-sectional studies [19] based on the following criteria: representativeness of the sample, sample size, ascertainment of exposure, and non-respondents, the subjects in different outcome groups are comparable, assessment of outcome, confounding factors are controlled. The adopted Newcastle–Ottawa scale score includes maximum of 10 points for cross-sectional studies and if the overall score was within 7–10 points (≥ 4 points in the section of selection, 1 point in the comparability section, ≥ 2 points in the section of exposure/outcome), the study is known as good quality [19].

## Results

### Selection of studies

**Fig. 1:**
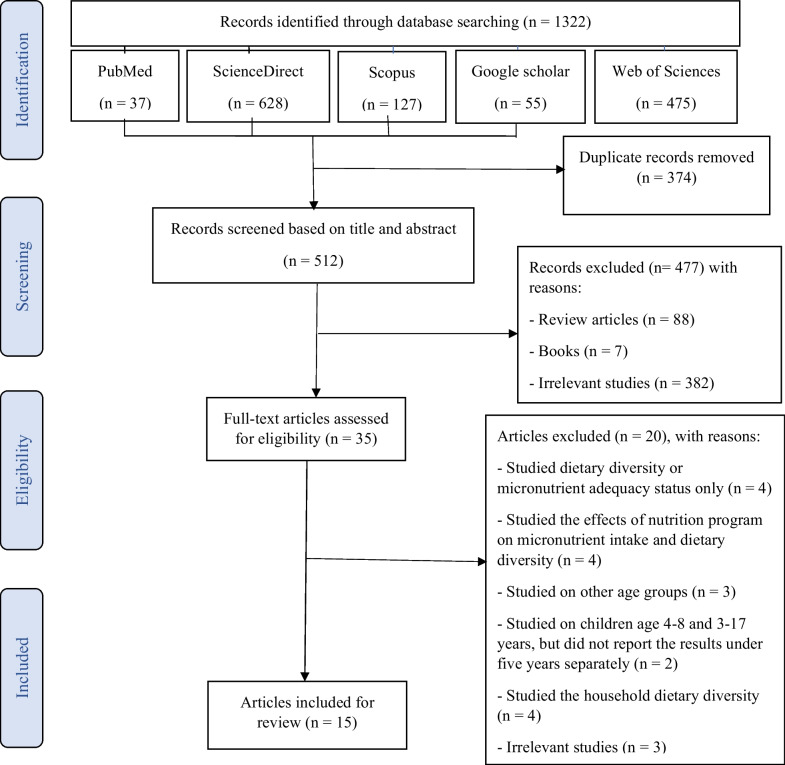
PRISMA diagram for the process of the search and study selection

In the initial search, 1322 records were found in electronic search databases including PubMed (*n* = 37), Scopus (*n* = 127), ScienceDirect (*n* = 628), Web of Sciences (*n* = 475), and Google Scholar (*n* = 55). After removing duplicates, 514 articles remained for further screening. Firstly, articles screened according to the title and abstract of those 477 studies were excluded for reasons: review articles (*n* = 88), book chapter (*n* = 7), and irrelevant studies (*n* = 384). Finally, the full text of the 35 remained articles was critically screened, of which 20 articles were excluded because they studied DD or micronutrient adequacy status only (*n* = 4), studied the effects of nutrition program on micronutrient intake and DD (*n* = 4), studied the household DD (*n* = 4), was on other age-groups (*n* = 3), studied on children aged 4–8 [20] and 3–17 [2] years, but did not report the results under 5 years separately (*n* = 2), and irrelevant studies (*n* = 3). No additional articles were found through forward and backward citation tracking of the eligible studies. Therefore, 15 studies that all had cross-sectional designs were included in this review (Tables 1, 2, and 3). PRISMA diagram for the process of the search and selection of this review is presented in Fig. 1.

**Table 1:** Characteristics of the studies that examined the relationship between dietary diversity and micronutrient adequacy in infants (under 2 years old)

First author (Year)	Country/study design	Sample size/age of infants	Method and duration of food intake assessment	No. of food groups considered/classification of DD	No. and type of micronutrients considered	DD criteria/MDA criteria	Adjusted covariates	Findings
Faber [21]	Africa/cross-sectional	316/6–24 months	24-h recall/2 days on different days of the week	7/Low DD: ≤ 3 groups	18/Thiamin, riboflavin, niacin, pantothenic acid, vitamin B6, folate, vitamin C, vitamin D, vitamin E, iron, zinc, magnesium, potassium, cupper, and calcium	WHO-UNICEF/WHO	–	High DD was associated with higher micronutrients density including calcium, iron, magnesium, potassium, phosphorus, zinc, riboflavin, niacin and vitamin D (*p* < 0.05)
				High DD: ≥ 4 groups				
Geng [22]	China/cross-sectional	1072/6–18 months	24-h and 7-d food recall/24 h	8/-	10/Thiamin, riboflavin, niacin, vitamin B6, vitamin B12, vitamin A, vitamin C, iron, zinc, and calcium	WHO/WHO	Age, weight, length, maternal education, mothers BMI and family income	There was significant association between NAR and DDS and FVS individually and when taken together (DDS + FVS) in all micronutrients except for niacin (*p* < 0.001)
Jones [23]	Bolivia/cross-sectional	251/6–23 months	24 h recall/24 h	7/6–8 months:	9/Thiamin, riboflavin, niacin, folate, vitamin C, vitamin A, iron, zinc, and calcium	WHO/-	Child age and sex, diarrhea symptoms in the previous two weeks, maternal height and education level, and household socioeconomic status	The 24 h food group diversity was positively associated with MMDA (*p* < 0.05)
				0 food groups = 0				
				1–2 food groups = 1				
				≥ 3 food groups = 2				
				9–11 months:				
				0 food groups = 0				
				1–2 food groups = 1				
				≥ 3 food groups = 2				
				12–23 months:				
				1 food groups = 0				
				2–3 food groups = 1 ≥ 4 food groups = 2				
Khor [24]	Malaysian/cross-sectional	119/6–23 months	24 h food record/2 days on different days of the week	7/MDD: ≥ 4 food groups	8/Thiamin, riboflavin, niacin, vitamin C, vitamin A, iron, zinc, and calcium	WHO/WHO	Breast-feeding status, minimum meal frequency, introduction of foods and minimum acceptable diet	The MDD had the greatest contribution to MAR [95% CI 3.09, 39.87 (*p* = 0.000)]
Mallard [25]	Zambian/cross-sectional	811/4–6 months	24-h recall/24 h	7/DDS: ranged 0–7	13/Thiamin, riboflavin, niacin, vitamin B6, vitamin B12, folate, vitamin A, vitamin C, iron, zinc, calcium, magnesium and phosphorus	WHO/-	Baseline hemoglobin concentration, birth weight, sex, HIV exposure, diarrhea, maternal height and education, and household wealth	MMDA was correlated with DD (*p* < 0.0001)
Moursi [26]	Madagascar/cross-sectional	702/6–23 months	24 h recall/24 h	7 and 8/DDS:	9/vitamin A, thiamin, riboflavin, vitamin B6, folate, vitamin C, calcium, iron, and zinc	-/ FAO-WHO	Child age, breast-feeding status, number of children, and nutritional status of child	DDS were strongly and positively associated with the MMDA (*p* < 0.05)
				0–8 for 8 food groups				
				0–7 for 7 food groups after excluding the fats and oils group				
				(a food group was counted only if at least 10 g was consumed)				
Wondafrash [27]	Ethiopia/cross-sectional	632/6–12 months	24 h recall/2 d	7/Good DD: ≥ 4 food groups	8/Vitamins A and C, thiamin, riboflavin, niacin, iron, calcium, and zinc	WHO/WHO	Socioeconomic index, child age, maternal age and schooling, diarrhea, cough, fever, sex and height-for-age Z -score	DDS was associated with the MMDA (*p* < 0·0001). A DDS of ≤ 2 food groups was the best predictor of ‘low’ MMDA

*CI* confidence interval, *DD* dietary diversity, *DDS* dietary diversity score, *FAO* Food and Agriculture Organization, *FVS* food variety score, *MDA* micronutrient density adequacy, *MMDA* mean micronutrient density adequacy, *MDD* minimum dietary diversity, *MDDS* minimum dietary diversity score, *MAR* mean adequacy ratio, *NAR* nutrient adequacy ratio, *UNICEF* United Nations International Children’s Emergency Fund, *WHO* World Health Organization

**Table 2:** Characteristics of the studies that examined the relationship between dietary diversity and micronutrient adequacy in children of 2–5 years old

First author (Year)	Country/study design	Sample size/age of infants	Method and duration of food intake assessment	No. of food groups considered/classification of DD	No. and type of micronutrients considered	DD criteria, MAR criteria	Adjusted covariates	Findings
Arsenault [28]	Bangladesh/cross-sectional	480/24–48 months	12-h weighed food records and 12-h recall/2 d	9/2 different DDS based on minimum amount of foods consumed	11/Thiamin, riboflavin, niacin, vitamin B6, folate, vitamin B12, vitamin C, vitamin A, iron, calcium, and zinc	–/WHO	Age, breast-feeding, stunted, wasted, season, and housing quality	MPA was positively associated with both DDS (*p* < 0.0001)
Diop [29]	Burkina Faso/cross-sectional	1066/24–59 months	24-h recall/24 h	11 and 7/MDD: ≥ 4 food groups	11/vitamin A, vitamin C, thiamin, riboflavin, niacin, vitamin B6, vitamin B12, folate, calcium, zinc, and iron	FAO and WHO/WHO	Energy intake	Both FGS-10 and FGS-7 had a positive linear association with MPAs (*p* < 0.001)
Kennedy [30]	Philippine/cross-sectional	3164/24–71 months	24-h recall/24 h	9/DDS: ranged 0–9	11/Thiamin, riboflavin, niacin, vitamin B6, folate, vitamin B12, vitamin C, vitamin A, iron, calcium, and zinc	–/WHO	Child age, sex, weight, and height, and energy intake	DDS was significant predictors of adequate micronutrient intake (*p* < 0.001)
Torrico [31]	Philippines/cross-sectional	1262/3–5 years	24-h recall/24 h	10/-	6/Iron, vitamin A, vitamin C, thiamin, riboflavin, and niacin	FAO/PDRI 2015	–	There was significant positive correlation between MAR and DDS (*r* = 0.29; *p* < 0.0001)
Zhao [32]	China/cross-sectional	1694/3–6 years	24-h dietary record/24 h	10/DDS ranged 0 to 9. Consuming at least 10 g from a unique food group except for the group of oils and fats	11/Thiamin, riboflavin, niacin, vitamin B6, folate, vitamin B12, vitamin C, vitamin A, iron, calcium, and zinc	FAO/FAO	Energy intake	DDS was positively correlated with indicators of micronutrient adequacy (*p* < 0.05)

*DD* dietary diversity, *DDS* dietary diversity score, *FAO* Food and Agriculture Organization, *FGS* food group score, *FVS* food variety score, *MAR* mean adequacy ratio, *MPA* mean probability of adequacy, *NAR* nutrient adequacy ratio, *PDRI 2015* Philippine Dietary Reference Intakes 2015, *WHO* World Health Organization

**Table 3:** Characteristics of the studies that examined the relationship between dietary diversity and micronutrient adequacy in children under 5 years old

First author (Year)	Country/study design	Sample size/age of infants	Method and duration of food intake assessment	No. of food groups considered/classification of DD	No. and type of micronutrients considered	DD criteria, MAR criteria	Adjusted covariates	Findings
Bekele [33]	Ethiopia/cross-sectional	538/6–59 months	24-h recall/one week	7/Good DD: ≥ 4 food groups	9/Thiamin, riboflavin, vitamin B6, folate, vitamin C, vitamin A, iron, calcium, and zinc	WHO/FAO	–	DDS ≥ 4 provided better predictions of MAR with 80.8% sensitivity and 45.8% specificity, and 60% correct classifications
Steyn [34]	Africa/cross-sectional	795/1–3 years 861/4–6 years	24-h recall/24 h	9 food groups for assessment DDS and 45 items for determination of FVS	11/Thiamin, riboflavin, niacin, vitamin B6, folate, vitamin B12, vitamin C, vitamin A, iron, calcium, and zinc	FAO/WHO-FAO	Energy intake	There was a high correlation between MAR and both FVS and DDS (*p* = 0.0001)
Steyn [35]	Africa/cross-sectional	795/1–3 years 861/4–6 years	24-h recall/24 h	6, 9, 13, and 21/-	11/Thiamin, riboflavin, niacin, vitamin B6, folate, vitamin B12, vitamin C, vitamin A, iron, calcium, and zinc	FAO/WHO-FAO	Energy intake	DDS based on 6, 9, 13, and 21 food groups was associated with MAR (*p* < 0.0001)

*DD* dietary diversity, *DDS* dietary diversity score, *FAO* Food and Agriculture Organization, *FGS* food group score, *FVS* food variety score, *MAR* mean adequacy ratio, *MPA* mean probability of adequacy, *NAR* nutrient adequacy ratio, *PDRI 2015* Philippine Dietary Reference Intakes 2015, *WHO* World Health Organization

### Quality of the articles

As shown in Additional file 2: Table S2, the mean quality score for cross-sectional studies was 9 out of 10. Thus, the majority of the studies had satisfactory scores, since, most of them used the appropriate methods for the recruitment of participants and included large sample sizes that were representative of the average in the wide community. Also, most of the included studies used the WHO guidelines for the definition of DD and MDA and controlled the potentially important confounders.

### Characteristics of the included studies

Because in some articles data were limited to only infants and in some only to preschoolers and in some others to under 5 years, the results were presented in three categories, accordingly. As presented in Tables 1, 2, and 3, around 53% of the included studies used WHO guidelines for the definition of DD and MDA and about 26% of the studies used Food and Agriculture Organization (FAO) guidelines. According to WHO guidelines, the consumption of four or more food groups of seven food groups was defined as minimum dietary diversity (MDD) or good DD. In order to determine the nutrient adequacy of the diet, the nutrient adequacy ratio is the ratio of a subject’s nutrient intake to the estimated average requirement calculated by the FAO/WHO recommended micronutrient (vitamins A, B6, B12, and C, niacin, thiamin, riboflavin, folate, calcium, iron, and zinc) intakes for children, was calculated for each of micronutrients. Nutrient intakes were assessed as inadequate, fairly adequate, and adequate intake if NAR < 66%, NAR = 66% to < 100%, and NAR ≥ 100%, respectively. The mean adequacy ratio (MAR) was calculated as the sum of NARs for all evaluated micronutrients divided by the number of micronutrients evaluated, expressed as a percentage. MAR ≥ 75% was considered as an adequate intake of micronutrients. For both NAR and MAR, a value of 100% is ideal because it shows that the intake is the same as the requirement. Other indicators of micronutrient adequacy including the probability of adequacy (PA), mean probability of adequacy  (MPA), MDA, and mean micronutrient density adequacy (MMDA) also were obtained by similar methods. Hence, NAR, MAR, PA, MPA, MDA, and MMDA refer to individual micronutrients level adequacy.

Above 57% of the studies were conducted in low-income countries and the studies were from both rural and urban regions. Results of the multivariate analysis were considered to evaluate the relation of the DDS with micronutrient adequacy, in this review. Except for one, all of the studies considered covariates such as age, sex, and energy intake in the analysis.

### Relationship of DD with micronutrient adequacy in infants (under 2 years old)

As presented in Table 1, totally, seven studies evaluated the relationship of DD with micronutrient adequacy in infants. All the studies reported that DD was positively associated with micronutrient adequacy.

### Relationship of DD with micronutrient adequacy in children of 2–5 years old

Totally, five studies evaluated the relationship between DD and micronutrient adequacy in children of 2–5 years. All the studies showed that high DD was associated with micronutrient adequacy in children (Table 2).

### Relationship of DD with micronutrient adequacy in children under 5 years

Totally, three studies evaluated the relationship between DD and micronutrient adequacy in children under 5 years. All the studies showed that high DD was associated with micronutrient adequacy in children (Table 3).

## Discussion

All the included studies reported a significant positive association between dietary diversity scores (DDS) with dietary micronutrients adequacy. Several reasons are involved in these positive associations, individuals with higher DD have more access to a variety of foods; therefore, they have higher food consumption and nutrient intake and also it reflects the more consumption of higher nutrient density and healthy foods, for example, fruit, vegetables, and whole grains, which could explain the higher intake of micronutrients [36].

In the study by Meng et al., food variety scores of fruits and vegetables and food variety scores of animal foods were positively correlated with overall micronutrient adequacy and NAR for most micronutrients. Also subjects with the high food variety scores of fruits and vegetables and food variety scores of animal foods had a lower risk of inadequate intake for most micronutrients [2]. This makes perfect sense because fruits and vegetables are rich in vitamins and minerals [37] such as carotene, potassium, vitamin C, vitamin B family, and vitamin D [38] and animal foods are an excellent source of several minerals and vitamins like magnesium, zinc, iron, calcium, and vitamins E and B [2]. Faber et al. demonstrated that more than 85% of children consumed ‘Cereals and roots/tubers’ in both rural and urban areas that less than 25% of them achieved the MDD. Diets with low micronutrient levels probably reflect the infrequent intake of fruits and vegetables as well as of foods of the animal origin [26]. Consuming more food groups will ensure an adequate intake of vitamins, especially vitamin B12 and vitamin C. But for minerals such as zinc and calcium, consuming more food groups barely affects the dose of absorption, thus we should pay attention to the lower bioavailability of minerals. Diet mostly based on plants is seldom enough to provide balanced nutrients because there are chemical substances in plants such as phytate, oxalate, and polyphenols that obstruct the absorption of minerals in the alimentary canal [39, 40]. Since deficiency of main nutrients in a basic stage of the lifecycle will be carried to the next stage in the life cycle [41], the WHO to improve micronutrient intake recommended the intake of nutrient-rich foods such as animal foods or foods fortified with micronutrients [42].

Lack of DD is a considerable problem among poor populations in the developing world because their diets are predominantly of starchy staples and few fresh fruits and vegetables with little or no animal products [43]. In the present study, 50% of the included studies were conducted in low-income countries; in the majority of them, starchy foods were the predominant staple food of children. The diets based on plants will lead to inadequate intake of mineral micronutrients and poor absorption [44]. Increasing DD and food variety can enhance the dietary intake of micronutrients. High DD also is associated with positive outcomes such as high nutrient intake, improved hemoglobin levels, improved child anthropometric measurements, and reduced mortality [30]. At all, the intake of nutrients below the recommended level of intake can result in deficiency without or with discernable clinical signs and therefore potential adverse nutritional and health consequences [45]. Elevating the diversification of diets is one of the main international strategies for improvement in the micronutrient intake and status [26]. Nevertheless, in developing countries because there is a frequent food insecurity problem, this is a difficult option for households. Furthermore, improving DD through agricultural biodiversity, specifically in the rural areas, could make an important contribution to improvements in nutritional outcomes [45].

Employing a large sample size and using valid guidelines for the definition of DD and micronutrients adequacy by the included studies were strengths of this review. Nevertheless, all of the included studies were cross-sectional and administrated in diverse seasons.

## Conclusion

All of the studies included in this systematic review revealed that high DD was significantly associated with micronutrients adequacy in infants and children under 5 years old. Therefore, the findings of the current study indicate that DD could be used for screening purposes to identify the risk of micronutrient inadequacy in children. This study recommends promoting the consumption of a diversified diet to achieving adequate micronutrients by children. Enhancing agricultural biodiversity and promoting maternal nutritional information that leads to choosing the correct and various food groups can increase the DDS of children under 5 years. Also, in low-income households, if the intake of various food groups is impossible, micronutrients supplements should be used for children to attain adequate micronutrients that are required for their rapid growth and development.

## Supplementary Information

**Additional file 1: Table S1.** Does the high dietary diversity score predict dietary micronutrients adequacy in children under 5 years old? A systematic review: Method of the database search strategy using PubMed, Scopus, ScienceDirect, Google Scholar, and Web of Sciences

**Additional file 2: Table S2.** Newcastle–Ottawa scale for assessment of quality of 15 included cross-sectional studies evaluating the relationship between dietary diversity and micronutrient adequacy

## Data Availability

All data included in this systematic review are from previously published papers.

## Abbreviations

MND: Micronutrient deficiency
WHO: World Health Organization
DD: Dietary diversity
MDA: Micronutrient density adequacy
MDD: Minimum dietary diversity
MAR: Mean adequacy ratio
DDS: Dietary diversity score
FAO: Food and Agriculture Organization
PA: Probability of adequacy
MMDA: Mean micronutrient density adequacy
