# Supplementary material for: Does the high dietary diversity score predict dietary micronutrients adequacy in children under 5 years old? A systematic review
Source: J Health Popul Nutr. 2023 Jan 6;42:2. doi: 10.1186/s41043-022-00337-3 (PMC9817313; doi:10.1186/s41043-022-00337-3)
Supplement: Supplementary file 1 — Additional file 1: Table S1. Does the high dietary diversity score predict dietary micronutrients adequacy in children under 5 years old? A systematic review: Method of the database search strategy using PubMed, Scopus, ScienceDirect, Google Scholar, and Web of Sciences [file 41043_2022_337_MOESM1_ESM.docx]

**Table 1 Supplementary.** Does the high dietary diversity score predict dietary micronutrients adequacy in children under five years old? A systematic review: Method of the database search strategy using PubMed, Scopus, ScienceDirect, Google Scholar, and Web of Sciences

| **Database (Search**  **conducted up to**  **February, 2022)** | **Search terms^a^** | **Number of studies searched** |
| --- | --- | --- |
| PubMed | 1# ((((((("dietary diversity"[Title/Abstract]) OR ("diet diversity"[Title/Abstract])) OR ("food diversity"[Title/Abstract])) OR ("dietary variety"[Title/Abstract])) OR ("diet variety"[Title/Abstract])) OR ("food variety"[Title/Abstract])) AND (micronutrient*[Title/Abstract])) AND (infant [Title/Abstract])  2# ((((((("dietary diversity"[Title/Abstract]) OR ("diet diversity"[Title/Abstract])) OR ("food diversity"[Title/Abstract])) OR ("diet variety"[Title/Abstract])) OR ("dietary variety"[Title/Abstract])) OR ("food variety"[Title/Abstract])) AND (micronutrient*[Title/Abstract])) AND (children [Title/Abstract])  1# & 2# (((((((("dietary diversity"[Title/Abstract]) OR ("diet diversity"[Title/Abstract])) OR ("food diversity"[Title/Abstract])) OR ("dietary variety"[Title/Abstract])) OR ("diet variety"[Title/Abstract])) OR ("food variety"[Title/Abstract])) AND (micronutrient*[Title/Abstract])) AND (infant[Title/Abstract])) AND (((((((("dietary diversity"[Title/Abstract]) OR ("diet diversity"[Title/Abstract])) OR ("food diversity"[Title/Abstract])) OR ("diet variety"[Title/Abstract])) OR ("dietary variety"[Title/Abstract])) OR ("food variety"[Title/Abstract])) AND (micronutrient*[Title/Abstract])) AND (children[Title/Abstract])) | 45  166  37 |
| SCOPUS | #1 (TITLE-ABS-KEY ("dietary diversity") OR TITLE-ABS-KEY ("diet diversity") OR TITLE-ABS-KEY ( "food diversity" ) OR TITLE-ABS-KEY ( "diet variety" ) OR TITLE-ABS-KEY ( "dietary variety" ) OR TITLE-ABS-KEY ( "food variety" ) AND TITLE-ABS-KEY ( "micronutrient*" ) AND TITLE-ABS-KEY ( children ) )  #2 (TITLE-ABS-KEY ("dietary diversity" ) OR TITLE-ABS-KEY ( "diet diversity" ) OR TITLE-ABS-KEY ( "food diversity" ) OR TITLE-ABS-KEY ( "diet variety" ) OR TITLE-ABS-KEY ( "dietary variety" ) OR TITLE-ABS-KEY ( "food variety" ) AND TITLE-ABS-KEY ( "micronutrient*" ) AND TITLE-ABS-KEY ( infant ) )  #1 & #2 ( ( TITLE-ABS-KEY ( "dietary diversity" ) OR TITLE-ABS-KEY ( "diet diversity" ) OR TITLE-ABS-KEY ( "food diversity" ) OR TITLE-ABS-KEY ( "diet variety" ) OR TITLE-ABS-KEY ( "dietary variety" ) OR TITLE-ABS-KEY ( "food variety" ) AND TITLE-ABS-KEY ( "micronutrient*" ) AND TITLE-ABS-KEY ( infant ) ) ) AND ( ( TITLE-ABS-KEY ( "dietary diversity" ) OR TITLE-ABS-KEY ( "diet diversity" ) OR TITLE-ABS-KEY ( "food diversity" ) OR TITLE-ABS-KEY ( "diet variety" ) OR TITLE-ABS-KEY ( "dietary variety" ) OR TITLE-ABS-KEY ( "food variety" ) AND TITLE-ABS-KEY ( "micronutrient*" ) AND TITLE-ABS-KEY ( children ) ) ) | 250  134  127 |
| Google Scholar | allintitle: "dietary diversity" AND “micronutrient”  allintitle: "diet diversity" AND “micronutrient”  allintitle: "food diversity" AND “micronutrient”  allintitle: "dietary variety" AND “micronutrient”  allintitle: "diet variety" AND "micronutrient"  allintitle: "food variety" AND "micronutrient"  Total | 41  6  2  4  0  2  55 |
| ScienceDirect | Title, abstract, keywords: "dietary diversity" OR " diet diversity" OR "food diversity" OR "dietary variety" OR "diet variety" OR "food variety" AND "micronutrient" | 628 |
| Web of Sciences | #1 (TI=("dietary diversity" OR "diet diversity" OR "food diversity" OR "dietary variety" OR "diet variety" OR "food variety" AND "micronutrient*") ) AND LANGUAGE: (English) AND DOCUMENT TYPES: (Article)  #2 (AB=("dietary diversity" OR "diet diversity" OR "food diversity" OR "dietary variety" OR "diet variety" OR "food variety" AND "micronutrient*") ) AND LANGUAGE: (English) AND DOCUMENT TYPES: (Article)  #3 (AK=("dietary diversity" OR "diet diversity" OR "food diversity" OR "dietary variety" OR "diet variety" OR "food variety" AND "micronutrient*") ) AND LANGUAGE:(English) AND DOCUMENT TYPES: (Article)    #1 AND #2 AND #3 | 818  2813  1030  475 |
| Total |  | 1322 |

^a^Searches were limited to original articles, and studies published in the English language using the appropriate filters and/or search terms depending on the database.
