# Supplementary material for: Does the high dietary diversity score predict dietary micronutrients adequacy in children under 5 years old? A systematic review
Source: J Health Popul Nutr. 2023 Jan 6;42:2. doi: 10.1186/s41043-022-00337-3 (PMC9817313; doi:10.1186/s41043-022-00337-3)
Supplement: Supplementary file 2 — Additional file 2: Table S2. Newcastle–Ottawa scale for assessment of quality of 15 included cross-sectional studies evaluating the relationship between dietary diversity and micronutrient adequacy [file 41043_2022_337_MOESM2_ESM.docx]

**Table 2 Supplementary.** Newcastle-Ottawa scale for assessment of quality of 15 included cross-sectional studies evaluating the relationship between dietary diversity and micronutrient adequacy

| First author  (Year) | Quality assessment criteria | | | | | | | | | | | | | | | | | | | | Overall Quality Score (Maximum = 10) |
| --- | --- | --- | --- | --- | --- | --- | --- | --- | --- | --- | --- | --- | --- | --- | --- | --- | --- | --- | --- | --- | --- |
|  | **Selection** | | | | | | | | | | | | **Comparability** | | **Outcomes** | | | | | |  |
|  | **Representativeness of the sample** | | | | **Sample size** | | **Non-respondents** | | | **Ascertainment of the exposure** | | |  |  | **Ascertainment of the outcome** | | | | **Statistical test** | |  |
|  | Truly representative of the average in the target population | Somewhat representative of the average in the target population | Selected group of users | No description of the sampling strategy | Justified and satisfactory | Not justified | Comparability between respondents and non-respondents characteristics is established, and the response rate is satisfactory | The response rate is unsatisfactory, or the comparability between respondents and non-respondents is unsatisfactory | No description of the response rate or the characteristics of the responders and the non-responders | Validated measurement tool | Non-validated measurement tool, but the tool is available or described | No description of the measurement tool | The study controls for the most important factor (energy intake) | The study control for any additional factor | Independent blind assessment | Record linkage | Self-report | No description | The statistical test used to analyze the data is clearly described and appropriate, and the measurement of the association is presented | The statistical test is not appropriate, not described or incomplete |  |
| Arsenault  (2012) [39] | * |  |  |  | * |  | * |  |  |  | * |  |  | * | ** |  |  |  | * |  | 8 |
| Bekele  (2020) [40] | * |  |  |  | * |  | * |  |  | ** |  |  | NS | NS | ** |  |  |  | * |  | 8 |
| Diop  (2021) [42] | * |  |  |  | * |  | * |  |  | ** |  |  | * |  |  |  |  | * | * |  | 8 |
| Faber  (2016) [33] | * |  |  |  |  | - | * |  |  | ** |  |  | NS | NS | ** |  |  |  | * |  | 7 |
| Geng  (2018) [34] | * |  |  |  | * |  | * |  |  | ** |  |  |  | * | ** |  |  |  | * |  | 9 |
| Jones  (2015) [35] | * |  |  |  |  | - | * |  |  | ** |  |  |  | * |  | * |  |  | * |  | 7 |
| Kennedy  (2007) [31] | * |  |  |  | * |  | * |  |  |  | * |  | * |  | ** |  |  |  | * |  | 8 |
| Khor  (2016) [36] | * |  |  |  |  | - | * |  |  | ** |  |  |  | * | ** |  |  |  | * |  | 8 |
| Mallard  (2016) [37] | * |  |  |  | * |  | * |  |  | ** |  |  |  | * |  | * |  |  | * |  | 8 |
| Moursi  (2008) [24] | * |  |  |  | * |  | * |  |  |  | * |  |  | * | ** |  |  |  | * |  | 8 |
| Steyn  (2006) [42] | * |  |  |  | * |  | * |  |  | ** |  |  | * |  | ** |  |  |  | * |  | 9 |
| Steyn  (2014) [43] | * |  |  |  | * |  | * |  |  | ** |  |  | * |  | ** |  |  |  | * |  | 9 |
| Torrico  (2021) [45] | * |  |  |  | * |  | * |  |  |  | * |  | NS |  | ** |  |  |  | * |  | 7 |
| Wondafrash  (2016) [38] | * |  |  |  | * |  | * |  |  | ** |  |  |  | * | ** |  |  |  | * |  | 9 |
| Zhao  (2017) [44] | * |  |  |  | * |  | * |  |  | ** |  |  | * |  |  | ** |  |  | * |  | 9 |

*, Acceptable; NS, not stated
